# An Isolated Perfused Rat Liver Model: Simultaneous LC-MS Quantification of Pitavastatin, Coproporphyrin I, and Coproporphyrin III Levels in the Rat Liver and Bile

**DOI:** 10.1021/acsomega.4c00109

**Published:** 2024-04-18

**Authors:** Nihan Izat, Ozan Kaplan, Mustafa Çelebier, Selma Sahin

**Affiliations:** † Department of Pharmaceutical Technology, Hacettepe University Faculty of Pharmacy, Ankara 06800, Turkey; ‡ Department of Analytical Chemistry, Hacettepe University Faculty of Pharmacy, Ankara 06100, Turkey

## Abstract

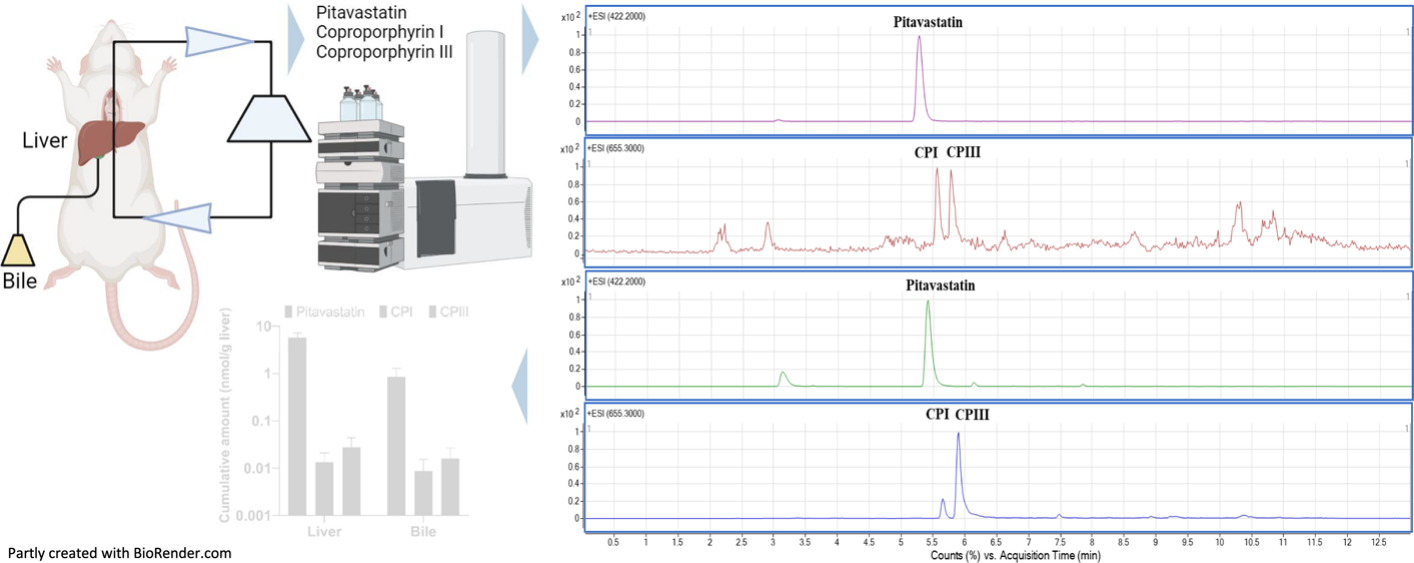

The isolated perfused rat liver (IPRL) model provides a mechanistic understanding of the organic-anion-transporting polypeptide (OATP/Oatp)-mediated pharmacokinetics in the preclinical evaluation, which often requires the use of control substrates (i.e., pitavastatin) and monitoring endogenous biomarkers (coproporphyrin I and III). This study aimed to develop and validate an LC-MS method allowing the simultaneous quantification of pitavastatin, coproporphyrin I (CPI), and coproporphyrin III (CPIII) in rat liver perfusion matrices (perfusate, liver homogenate, bile). The analysis was performed on a C18 column at 60 °C with 20 μL of sample injection. The mobile phases consisted of water with 0.1% formic acid and acetonitrile with 0.1% formic acid with a gradient flow of 0.5 mL/min. The assay was validated according to the ICH M10 Bioanalytical Method Validation Guideline (2022) for selectivity, calibration curve and range, matrix effect, carryover, accuracy, precision, and reinjection reproducibility. The method allowing the simultaneous quantification of pitavastatin, CPI, and CPIII was selective without having carryover and matrix effects. The linear calibration curves were obtained within various calibration ranges for three analytes in different matrices. Accuracy and precision values fulfilled the required limits. After 60 min perfusion with pitavastatin (1 μM), the cumulative amounts of pitavastatin in the liver and bile were 5.770 ± 1.504 and 0.852 ± 0.430 nmol/g liver, respectively. CPIII was a more dominant marker than CPI in both liver (0.028 ± 0.017 vs 0.013 ± 0.008 nmol/g liver) and bile (0.016 ± 0.011 vs 0.009 ± 0.007 nmol/g liver). The novel and validated bioanalytical method can be applied in further IPRL preparations investigating Oatp-mediated pharmacokinetics and DDIs.

## 1 Introduction

The human organic-anion-transporting polypeptides OATP1B1 and OATP1B3 expressed in the liver mediate the influx of endogenous and exogenous substrates into the hepatocytes. Due to the inhibition and/or induction of these drug transporters, clinical drug–drug interactions (DDIs) may occur. Thus, investigational drugs should be evaluated whether they are substrate/perpetrator of these drug transporters.^1−4^ Transporter-mediated DDI evaluations are commonly evaluated using model substrates and inhibitor(s) with the drug of interest. Pitavastatin, an antihyperlipidemic drug,^5,6^ is a well-known *in vitro* and clinical model substrate of human OATP1B1/3^7^ due to its high sensitivity toward OATP1B inhibition.^8−10^ Pitavastatin is also preferable in preclinical studies on rats as it demonstrates a selective liver distribution^11^ and has an affinity to rat Oatp1b2.^12^

More recently, various endogenous biomarkers in humans^13−19^ and preclinical species (e.g., rats, mice, cynomolgus monkeys)^20−22^ to evaluate their use in monitoring of drug transporters. Coproporphyrin I (CPI) and coproporphyrin III (CPIII) are anionic byproducts of heme biosynthesis, circulating in the blood and undergoing biliary and renal excretion. In Oatp-expressing HeLa cells, CPI was found a substrate of rodent Oatp1b2 whereas CPIII was a dual substrate of both Oatp1b2 and Oatp2b1.^22^ In a study using membrane vesicles, the Mrp2 transporter was found to be active in CPI transport.^25^ Bezençon et al. showed altered disposition of CPs by loss of Mrp2 and increased Mrp3 function in a rat model.^22^ Comparing the wild type with the knockout (Oatp1*a*/1b^–/–^) mice, CPI and CPIII were found to be Oatp1*a*/1b substrates.^20^

For the preclinical evaluation of Oatp-mediated pharmacokinetics and related DDIs, rat hepatocytes or isolated perfused rat liver (IPRL) preparations have been widely used.^26−30^ The IPRL assay is a well-established model^31^ and enables the collection of the perfusion solution, liver tissue, and bile samples, thus providing a mechanistic understanding of hepatic drug disposition^31−35^ while monitoring endogenous biomarkers throughout the experiment.

CPI and CPIII are considered as Tier 1 and exploratory endogenous biomarkers for hepatic OATP1B1 and OATP1B3, respectively^23,24^ because they are metabolically stable, and their basal level is not dependent on race or circadian rhythm.^22^ As a Tier 1 biomarker, CPI is recommended to be monitored in plasma in early-phase clinical studies, considering selectivity, sensitivity, and prediction performance. The function of OATPs also affects the concentration of their substrates in the liver tissue, which is not routinely collected in clinical studies, as it requires invasive tissue biopsy. However, as a preclinical tool, the IPRL model allows the mechanistic investigation of the hepatobiliary disposition of probe OATP substrates (e.g., pitavastatin) and endogenous biomarkers (e.g., CPI and III) in the presence and absence of other investigational drugs, which may inhibit OATPs and cause transporter-mediated DDIs.

This study aimed to provide a novel liquid chromatography coupled with mass spectrometry (LC-MS) method for the simultaneous quantification of pitavastatin, CPI, and CPIII in the liver tissue and bile and validate it according to the current International Council for Harmonisation of Technical Requirements for Pharmaceuticals for Human Use (ICH) M10 Guideline (2022)^36^ and apply it to the samples obtained from the IPRL model, investigating the hepatobiliary disposition of pitavastatin, CPI, and CPIII.

## 2 Materials and Methods

### 2.1 Chemicals

Pitavastatin calcium and CPIII dihydrochloride were obtained from Abcam (UK) and Chemische Fabrik Berg (Germany), respectively. CPI dihydrochloride, bovine serum albumin (BSA), acetonitrile (LC-MS grade), formic acid solution, and sodium taurocholate were purchased from Sigma-Aldrich (USA). Water was purified by using a Milli-Q system (Millipore, USA). All other chemicals were of analytical grade and were obtained commercially.

### 2.2 Animals

Male Sprague–Dawley rats (*n* = 6; Kobay Laboratories, Turkey) were handled according to the approved study protocol by the Hacettepe University Animal Experimentations Ethics Board. Rats were randomly divided into two groups to undergo *in situ* liver perfusion by either blank buffer (*n* = 3; body weight: 305.60 ± 9.20 g; wet liver weight: 7.99 ± 1.88 g) or pitavastatin containing buffer (*n* = 3; body weight: 261.82 ± 16.41 g; wet liver weight: 8.25 ± 1.61). They were kept under a 12 h light–dark cycle in a temperature-controlled environment and fed on a standard laboratory diet with free access to water.

### 2.3 *In Situ* Isolated Perfused Liver Studies

The *in situ* isolated perfused rat liver study was performed to collect biological matrices. Under intraperitoneal anesthesia (ketamine: 90 mg/kg and xylazine: 5 mg/kg), a polyethylene tubing (o.d. 0.61 mm, i.d. 0.28 mm) was used to cannulate the bile duct and bile was collected cumulatively until the end of the experiment. The portal vein and thoracic vena cava were cannulated using a 16GA catheter (Argyle Medicut, o.d. 1.7 mm × 45 mm) and a 14GA catheter (Argyle Medicut, o.d. 2.1 mm × 45 mm), respectively, and all loose ligatures were tied securely. Through a connection tubing, the liver was perfused (15 mL/min) through the portal vein in a recirculatory manner from a reservoir containing 150 mL of perfusate saturated with carbogen (95% O_2_/5% CO_2_) and composed of Krebs bicarbonate buffer (pH 7.4, 37 °C) containing 3 g/L glucose, 6 mg/L sodium taurocholate, and 1% BSA. During the perfusion procedure, the liver was moistened with the perfusion buffer and covered with a piece of parafilm to minimize evaporation. The liver was cleared from the blood within a stabilization period of 20 min. Afterward, the reservoir was replaced with the fresh blank perfusate buffer (for standard preparation) or 1 μM pitavastatin in perfusate buffer (for sample preparation). After 60 min of perfusion, the circulated perfusate and bile were collected. The liver was removed, blotted dry, and weighed, and its homogenate was prepared immediately with ice-cold Krebs bicarbonate buffer in a (v/v) 1:2 ratio using a homogenizer (Isolab homogenizer, light duty, Isolab, Germany) at 18000 rpm. All samples were frozen at −20 °C before using them in the preparation of standard solutions. Amounts of pitavastatin, CPI, and CPIII in the liver and bile have been calculated and compared by the Mann–Whitney *U* test.

### 2.4 Preparation of Standard Solutions and Samples

To prevent the photodegradation of CPI/III, all solutions were prepared and stored in amber-colored tubes for 2 h. Stock solutions of pitavastatin, CPI, and CPIII in the circulated perfusate (containing 1% DMSO) were prepared at 20 μM. Spiking solutions were prepared from alternating stock solutions. The ranges for these spiking solutions were 0.25–20 μM pitavastatin and 0.01–2 μM CPI/III for the perfusate and liver. For perfusate standards, spiking solutions and 6 M formic acid (100 μL) were mixed in a 1:1 (v/v) ratio. For liver standards, spiking solutions, liver homogenate, and 6 M formic acid were mixed in a 1:1:2 (v/v) ratio.

For bile standards, stock solutions of pitavastatin and CPI/III were prepared in 6 M formic acid solution (1% DMSO) at 100 and 20 μM, respectively. They are used in alternating volumes to prepare spiking standards (10–100 μM pitavastatin and 1–20 μM CPI/III) and added into blank bile in a 1:1 (v/v) ratio.

For sample preparation, 100 μL of 6 M formic acid solution was added to 100 μL of perfusate and bile samples. Liver samples (100 μL) were first diluted with blank Krebs buffer (100 μL) and afterward acidified with 200 μL of 6 M formic acid solution.

After the agitation with a vortex (V1 Plus, Biosan, Lithuania) at 1400 rpm for 5 min, 1 mL of methyl tertiary butyl ether (mtbe) was added and the mixture was immediately centrifuged at 2325*g* for 10 min. After the collection of the supernatant (0.5 mL), this step was repeated for an additional two rounds and 1.5 mL of supernatant was collected from each tube. The organic solvent was evaporated within 30–60 min using the vacuum concentrator at 4 °C. After reconstitution in the mixture of acetonitrile and water (65:35 (v/v), 0.1% formic acid) samples, calibration standards and quality control samples (QCs) were obtained. The concentration ranges are given in Table 1.

**Table 1:** Calibration Standards and Quality Control Samples

	pitavastatin (μM)			CPI and CPIII (μM)	
QCs	**perfusate**	**liver**	**bile**	**liver**	**bile**
LLOQ	0.025	0.025	0.500	0.010	0.050
low	0.050	0.075	1.000	0.025	0.150
medium	0.075	0.250	2.500	0.100	0.300
	0.250	0.750	10.00	0.250	1.000
high	0.500	2.500	30.00	0.300	3.000
	1.500	5.000	50.00	0.500	5.000

### 2.5 Instrumentation and Chromatographic and Mass Spectrometric Conditions

An Agilent Technologies 6530 Accurate-Mass Q-TOF LC-MS system (USA) equipped with a Restek Raptor (USA) C18 column (150 × 4.6 mm i.d., 2.7 μm particle size) and a C18 guard column was used for LC-MS analysis following the method and sample preparation optimization. Twenty microliters of sample was injected into the column at 60 °C, and elution was achieved using a gradient flow at a rate of 0.5 mL/min. The mobile phases consisted of (A) water with 0.1% formic acid and (B) acetonitrile with 0.1% formic acid. The gradient was 35 to 90% B from 0 to 7 min and 90 to 35% B from 7 to 10 min and held at 35% B for 3 min. The dual electrospray ion source was operated in positive ion mode with an ion spray voltage of 4000 V and a source temperature of 350 °C. The nebulizer gas was 45 psi, and the drying gas was 10 L/min. The *m*/*z* ratios were 422.2 for pitavastatin and 655.3 for CPI/III. The system suitability test was performed considering the efficiency of the method (the number of theoretical plates) and the symmetry factor.

### 2.6 Bioanalytical Method Validation

Bioanalytical validation of the assay was performed according to the ICH M10 guideline (2022)^36^ based on selectivity, specificity, calibration curve and range, matrix effect, carryover, accuracy, precision, and reinjection reproducibility.

The selectivity of the analytical method was assessed by the responses of blank (drug-free) matrices (circulated perfusate, liver, and bile) acquired from three individual animals. As CPI and CPIII are endogenous molecules, only circulated perfusate was used as a blank matrix. To establish the specificity of the method for the simultaneous analysis of all three molecules (pitavastatin, 1 μM; CPI, 0.1 μM; CPIII, 0.1 μM), any interference was investigated in the presence of the other two. If there is an interfering substance, its response should be less than 20% of the analyte response at the LLOQ to ensure selectivity and specificity.

The calibration range was determined between the lower limit of quantification (LLOQ) and upper limit of quantification (ULOQ), which vary for different molecules and matrices, as given in Table 1. The LLOQ was the lowest standard that could be quantified within 20% of the nominal concentration and at which six replicates could be reproducible having less than 20% relative standard deviation (RSD). QCs were determined at four levels including LLOQ. The low QC was prepared to be close to the concentration of three times of LLOQ. The high QC was determined as 75% of the ULOQ, and the medium QC was selected within the calibration range. Calibration curves were obtained by plotting the peak areas against the corresponding nominal concentrations according to the linear regression model. The background subtraction approach was applied for CPs as they are endogenous molecules.^36^ The linearity of the curve was demonstrated by the calibration equation, which is characterized by determination coefficient, slope, intercept, and standard errors of slope and intercept.

The matrix effect was evaluated by analyzing low and high QC samples (*n* = 3), each prepared using the perfusate, liver, and bile matrices from three different animals. The percent relative error (RE) and the RSD values should be less than 15% to neglect the matrix effect. The carryover was assessed by injecting blank samples after analyzing the calibration standard at the ULOQ. It was considered negligible if the measured peak area in the blank samples was less than 20% of LLOQ. Within run and between run, accuracy and precision were evaluated by analyzing six replicates of each QC level over 3 days by three runs. The accuracy and precision of the method are assessed via RE and RSD, respectively. These values should be within ±20% at the LLOQ and within ±15% at all other calibration levels to ensure within-run and between-run accuracy and precision. The reproducibility of the analytical method was assessed by replicated measurements of the QCs and included in the accuracy and precision assessment.

## 3 Results

### 3.1 Optimization of LC/MS Conditions

**Figure 1:**
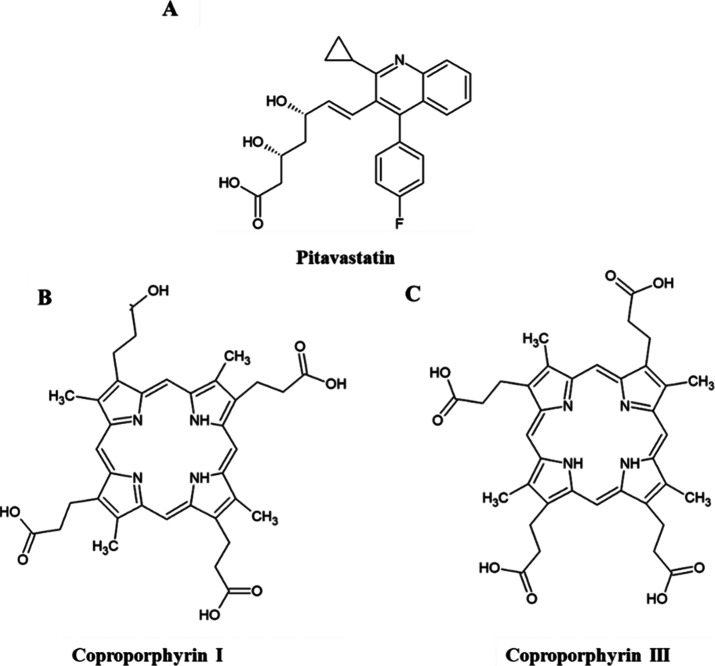
Molecular structures of (A) pitavastatin, (B) coproporphyrin I, and (C) coproporphyrin III. Molecules were drawn using ACD/ChemSketch.^38^

The successful concurrent analysis of pitavastatin, CPI, and CPIII was achieved once the method was optimized following the analyses in varied conditions. Based on the nonpolar structures (Figure 1) and physicochemical properties (logP is 3.75 and 5, for pitavastatin and CPI/III, respectively) of analytes, we selected reverse-phase chromatography using the C18 column.^37^

Early trials were done using a C18 column having a particle size of 1.7 μm (100 × 2.1 mm i.d), which resulted in back-pressure issues, causing the developed method hard to apply where HPLC devices exist. Considering the adaptability of the method in LC-MS, we attended to perform the analysis with columns having bigger particle sizes. Afterward, we achieved the best resolution with the Restek Raptor (USA) C18 column (150 × 4.6 mm i.d., 2.7 μm particle size) at 60 °C provided the best resolution with 20 μL sample injection. The mobile phases consisted of (A) water with 0.1% formic acid and (B) acetonitrile with 0.1% formic acid. The separation of CPI and CPIII peaks was not achieved (resolution <1.5)^36^ with the isocratic mode. Therefore, a linear gradient elution program was tested and applied. The two parameters, separation and elution time, were considered to find the optimum condition. When the gradient elution program was applied, as mentioned in Section 2, a gradient elution program between 4 and 7 min was capable of separating the targeted compounds with a shorter elution time. The final gradient elution program (35 to 90% B from 0 to 7 min and 90 to 35% B from 7 to 10 min and hold at 35% B for 3 min) at a rate of 0.5 mL/min was fixed to capture three analytes as separated and symmetrical peaks.

Detection sensitivity in mass spectrometers is directly related to ionization efficiency.^39^ In order to increase the ionization efficiency, optimum values were determined for parameters such as the drying gas temperature flow rate and ion source voltage. Ion source parameters were determined as gas temperature 350 °C, gas flow 10 L/min, and nozzle voltage 1000 V in positive ionization mode. Ionization efficiency is also linked to the ion suppression phenomenon that may occur in the sample matrix. Since the ionic characters and physical and chemical properties of the droplets formed in the spray change, the ionization efficiency also changes.^40^ At this point, the ion suppression effect and recovery of the targeted compounds were taken into consideration, and different sample preparation techniques such as solid phase extraction, liquid–liquid extraction, and precipitation with organic solvent were compared within each other. The method detailed in Section 3.2 was chosen as the optimum sample preparation method in which the extraction efficiency is maximum where the ion suppression effect does not interfere the analysis. The efficiency of the method in the analysis of samples in all matrices was more than 2000, and the symmetry factors were less than 2, confirming the suitability of the system (Supplemental Table 1).

### 3.2 Optimization of the Sample Preparation Method

Following the optimization of the method conditions, the sample preparation method was optimized to achieve maximum efficiency. Various options, including liquid–liquid extraction by single centrifuge or triple centrifuge, or using a liquid phase cartridge (Extrelut NT3, Sigma-Aldrich, USA), and solid phase extraction (Supelco Sigma-Aldrich, USA) using ethyl acetate and/or MTBE methods were evaluated. Analyte responses were evaluated based on the selectivity and recovery parameters. Solid phase extraction and liquid–liquid extraction by a single centrifuge and liquid cartridge with MTBE provided 1–17% recovery for CPs. Although the highest recovery was obtained with the method using liquid phase cartridges with ethyl acetate (54–64%), the liquid–liquid extraction (triple centrifuge) method (2 mL of MTBE in total for each sample) has been selected due to the lowest matrix interference to the signal peaks of the analytes and relatively high recovery (42–50%) compared the former methods.

### 3.3 Bioanalytical Method Validation

#### 3.3.1 Selectivity

**Figure 2:**
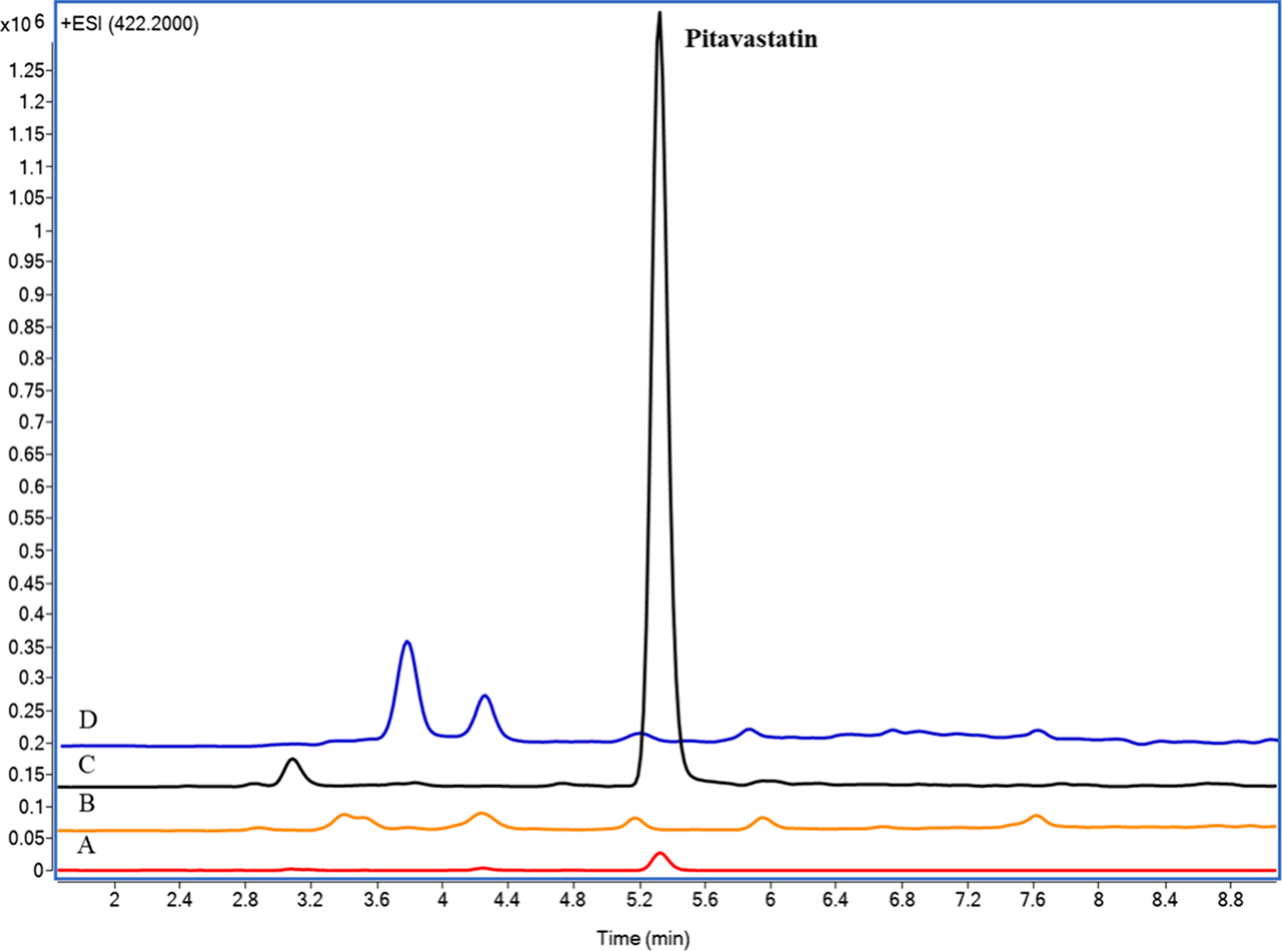
LC-MS chromatograms of blank perfusate (A), blank liver (B), pitavastatin (0.025 μM) in blank perfusate (C), and blank bile (D).

**Figure 3:**
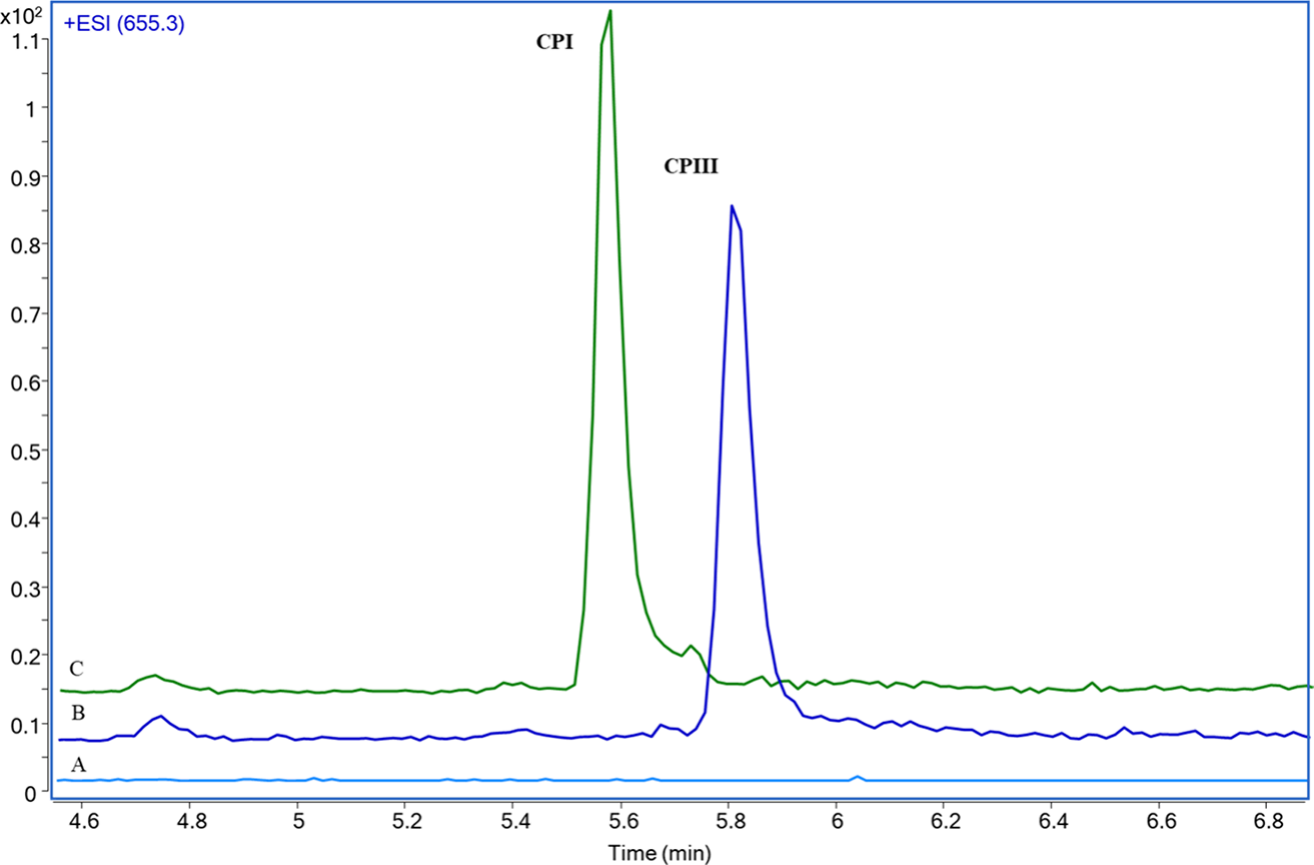
LC-MS chromatograms of blank perfusate (A), coproporphyrin III (CPIII; 0.1 μM) (B), and coproporphyrin I (CPI; 0.1 μM) (C) in blank perfusate.

Detected responses of blank perfusate, liver and bile at *m*/*z* of pitavastatin (422.2) were less than 20% of the analyte response at the LLOQ of each matrix condition (Figure 2). There were no interfering peaks at *m*/*z* of CPI/III (655.3) after the injection of blank perfusate, confirming that the method is selective for all compounds (Figure 3).

#### 3.3.2 Calibration Curve and Range

The calibration curves of pitavastatin in the range of 0.025–1.5 μM for the perfusate, 0.025–5 μM for the liver, and 0.5–50 μM for the bile matrices were linear, and the calibration equations were characterized by intercept, slope, and determination coefficient (*R*^2^) via a linear regression analysis (Table 2). The calibration curves and equations of CPI and CPIII were obtained by subtracting the basal level from the standard solutions prepared for the determination from the liver (0.01–0.5 μM) and bile (0.05–5 μM) samples. As shown in Supplemental Figures 1–3, statistically significant linear concentration–response relationships were obtained for both endogenous substances in both matrices.

**Table 2:** Linearity of the Method Quantifying Pitavastatin, Coproporphyrin I (CPI), and Coproporphyrin III (CPIII) (*n* = 6) in the Isolated Perfused Rat Liver Matrices

		slope	intercept	*R*^2^
perfusate	**pitavastatin**	18,240,000 ± 526,927	740,589 ± 344,943	0.9967
liver	**pitavastatin**	12,480,000 ± 110,659	–176,332 ± 255,080	0.9997
	**CPI**	4,982,000 ± 118,641	–14,548 ± 31,135	0.9977
	**CPIII**	3,374,000 ± 83705	–30,466 ± 21,967	0.9975
bile	**pitavastatin**	6,307,000 ± 207,732	1,138,000 ± 5,023,000	0.9953
	**CPI**	2,855,000 ± 111,234	–405,162 ± 269,097	0.9940
	**CPIII**	5,631,000 ± 249,677	–114,400 ± 604,018	0.9922

#### 3.3.3 Carryover

Following the injection of the ULOQ into the column, detected responses of blank matrices were lower than 20% of the analyte response at the LLOQ (5.30 ± 2.70% in the perfusate, 2.84 ± 0.82% in the liver, 1.23 ± 0.48% in the bile, mean ± SD, *n* = 3). Thus, the carryover effect on the analysis of pitavastatin was excluded in all matrices. The blank perfusate, applied consecutively after the ULOQ, showed no interference at the *m*/*z* of CPI/III (655.3), confirming there was no carryover effect.

#### 3.3.4 Matrix Effect

Components in the biological sample matrix can cause alterations of the analyte response at the LC-MS ion source, irrespective of the analyte recovery due to the extraction method in sample preparation. In this study, the interfering matrix effect was excluded since the accuracy was within ±15% of the nominal concentration and the precision was below 15% of RSD between individual matrix sources (the perfusate, liver, and bile for pitavastatin; the liver and bile for CPI/III; Table 3).

**Table 3:** Matrix Effect on the LC-MS Analysis of Pitavastatin, CPI, and CPIII (Mean ± SD; *n* = 3)^a^

		theoretical (μM)	obtained (μM)	RE (%)	RSD (%)
perfusate	**pitavastatin**	0.050	0.050 ± 0.006	0.15	12.81
		0.500	0.525 ± 0.023	5.00	4.29
liver	**pitavastatin**	0.075	0.066 ± 0.009	11.43	12.99
		2.500	2.426 ± 0.047	2.97	1.94
	**CPI**	0.025	0.024 ± 0.002	5.83	9.57
		0.300	0.298 ± 0.020	0.83	6.61
	**CPIII**	0.025	0.026 ± 0.003	5.22	11.94
		0.300	0.296 ± 0.013	1.42	4.56
bile	**pitavastatin**	1.000	0.983 ± 0.063	1.73	6.41
		30.000	29.469 ± 0.980	1.77	3.32
	**CPI**	0.150	0.139 ± 0.021	7.66	14.96
		3.000	3.213 ± 0.176	7.10	5.48
	**CPIII**	0.150	0.150 ± 0.018	0.09	11.75
		3.000	3.060 ± 0.150	2.01	4.91

a Abbreviations: CPI, coproporphyrin I; CPIII, coproporphyrin III; RE, relative error; RSD, relative standard deviation.

#### 3.3.5 Accuracy, Precision, and Reinjection Reproducibility

Accuracy, precision, and reinjection reproducibility results are given in Tables 4, 5, and 6. All results met the criteria as the RE and RSD values were less than 15% at each level (20% at the LLOQ), confirming the accuracy and precision of the method, respectively.

**Table 4:** Accuracy, Precision (Within-Run and Between-Run) and Reinjection Reproducibility of the Method for Pitavastatin Analysis in the Rat Perfusate, Liver, and Bile (Mean ± SD; *n* = 6)^a^

	perfusate				liver				bile			
	**theoretical (μM)**	**obtained (μM)**	**RE (%)**	**RSD (%)**	**theoretical (μM)**	**obtained (μM)**	**RE (%)**	**RSD (%)**	**theoretical (μM)**	**Obtained (μM)**	**RE (%)**	**RSD (%)**
within-run	0.025	0.022 ± 0.001	13.03	6.56	0.025	0.024 ± 0.001	3.39	3.50	0.500	0.530 ± 0.015	5.95	2.88
	0.050	0.053 ± 0.007	5.58	13.10	0.075	0.078 ± 0.002	4.58	2.33	1.000	1.035 ± 0.015	3.46	1.50
	0.250	0.238 ± 0.017	4.85	6.96	0.75	0.732 ± 0.016	2.46	2.17	10.000	10.662 ± 0.786	6.62	7.37
	0.500	0.546 ± 0.017	9.25	3.05	2.5	2.660 ± 0.152	6.39	5.73	30.000	31.400 ± 1.794	4.67	5.71
between-run	0.025	0.021 ± 0.002	15.93	7.34	0.025	0.024 ± 0.001	2.19	2.52	0.500	0.527 ± 0.016	5.33	2.95
	0.050	0.050 ± 0.003	0.48	5.95	0.075	0.066 ± 0.009	11.97	13.63	1.000	1.033 ± 0.014	3.34	1.35
	0.250	0.230 ± 0.004	8.16	1.88	0.75	0.694 ± 0.050	7.52	7.20	10.000	10.865 ± 0.613	8.66	5.64
	0.500	0.541 ± 0.022	8.29	4.13	2.5	2.525 ± 0.227	0.99	8.98	30.000	31.556 ± 2.062	5.187	6.53
reinjection	0.025	0.024 ± 0.003	3.67	14.05	0.025	0.027 ± 0.004	7.40	14.64	0.500	0.560 ± 0.033	11.91	5.88
	0.050	0.044 ± 0.006	12.78	13.55	0.075	0.067 ± 0.009	11.30	12.84	1.000	1.001 ± 0.039	0.11	3.89
	0.250	0.227 ± 0.021	9.29	9.34	0.75	0.753 ± 0.002	0.35	0.32	10.000	10.855 ± 0.191	8.55	1.76
	0.500	0.531 ± 0.015	6.21	2.85	2.5	2.378 ± 0.028	4.88	1.19	30.000	31.423 ± 0.988	4.74	3.14

a Abbreviations: CPI, coproporphyrin I; CPIII, coproporphyrin III; RE, relative error; RSD, relative standard deviation.

**Table 5:** Accuracy, Precision (Within-Run and Between-Run) and Reinjection Reproducibility of the Method for CPI Analysis in the Rat Perfusate, Liver, and Bile (Mean ± SD; *n* = 6)^a^

	liver				bile			
	**theoretical (μM)**	**obtained (μM)**	**RE (%)**	**RSD (%)**	**theoretical (μM)**	**obtained (μM)**	**RE (%)**	**RSD (%)**
within-run	0.010	0.009 ± 0.001	7.44	9.22	0.050	0.060 ± 0.002	19.26	3.64
	0.025	0.024 ± 0.004	4.18	15.55	0.150	0.134 ± 0.007	10.70	4.97
	0.100	0.108 ± 0.014	8.37	12.91	1.000	0.915 ± 0.067	8.55	7.38
	0.300	0.330 ± 0.037	10.01	11.24	3.000	3.249 ± 0.034	8.30	1.03
between-run	0.010	0.009 ± 0.001	7.08	9.52	0.050	0.058 ± 0.003	15.31	4.59
	0.025	0.025 ± 0.001	1.57	4.62	0.150	0.144 ± 0.019	4.23	13.05
	0.100	0.107 ± 0.003	6.69	2.77	1.000	0.916 ± 0.068	8.41	7.40
	0.300	0.337 ± 0.020	12.49	5.91	3.000	3.127 ± 0.188	4.23	6.01
reinjection	0.010	0.009 ± 0.001	7.08	9.52	0.050	0.056 ± 0.003	11.56	5.57
	0.025	0.025 ± 0.001	1.57	4.62	0.150	0.164 ± 0.002	9.26	1.45
	0.100	0.107 ± 0.003	6.69	2.77	1.000	0.988 ± 0.007	1.20	0.69
	0.300	0.337 ± 0.020	12.49	5.91	3.000	3.069 ± 0.187	2.31	6.08

a Abbreviations: CPI, coproporphyrin I; CPIII, coproporphyrin III; RE, relative error; RSD, relative standard deviation.

**Table 6:** Accuracy, Precision (Within-Run and Between-Run) and Reinjection Reproducibility of the Method for CPIII Analysis in the Rat Perfusate, Liver, and Bile (Mean ± SD; *n* = 6)^a^

	liver				bile			
	**theoretical (μM)**	**obtained (μM)**	**RE (%)**	**RSD (%)**	**theoretical (μM)**	**obtained (μM)**	**RE (%)**	**RSD (%)**
within-run	0.010	0.010 ± 0.000	0.17	4.81	0.050	0.053 ± 0.004	5.90	8.02
	0.025	0.028 ± 0.002	10.73	6.88	0.150	0.145 ± 0.019	3.28	12.82
	0.100	0.096 ± 0.005	4.05	5.43	1.000	0.924 ± 0.023	7.64	2.52
	0.300	0.306 ± 0.021	2.13	6.95	3.000	3.153 ± 0.018	5.09	0.59
between-run	0.010	0.009 ± 0.002	11.41	19.90	0.050	0.050 ± 0.010	0.08	19.13
	0.025	0.028 ± 0.002	10.20	8.67	0.150	0.148 ± 0.018	1.24	12.34
	0.100	0.097 ± 0.005	3.22	4.94	1.000	0.937 ± 0.048	6.30	5.15
	0.300	0.303 ± 0.010	0.96	3.38	3.000	3.114 ± 0.083	3.81	2.66
reinjection	0.010	0.009 ± 0.002	7.55	17.66	0.050	0.047 ± 0.006	6.41	13.81
	0.025	0.026 ± 0.002	2.52	6.52	0.150	0.152 ± 0.014	1.44	9.51
	0.100	0.104 ± 0.004	4.16	3.74	1.000	0.957 ± 0.034	4.33	3.56
	0.300	0.345 ± 0.019	15.05	5.64	3.000	3.084 ± 0.175	2.79	5.68

a Abbreviations: CPI, coproporphyrin I; CPIII, coproporphyrin III; RE, relative error; RSD, relative standard deviation.

### 3.4 Hepatic Disposition of Pitavastatin, CPI, and CPIII following *In Situ* Isolated Rat Liver Perfusion

**Figure 4:**
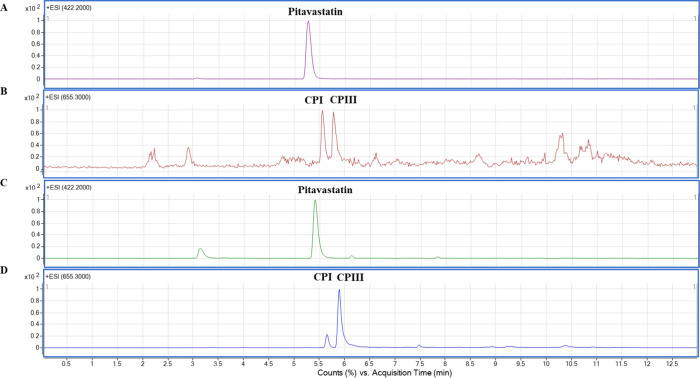
LC-MS chromatograms of pitavastatin and coproporphyrin I and III (CPI and CPIII) in liver homogenate (A, B) and bile samples (C, D) following 60 min perfusion of a rat liver with 1 μM pitavastatin.

The developed LC-MS method was successfully used for the simultaneous quantification of pitavastatin, CPI and CPIII in the liver and bile samples collected from *in situ* isolated rat liver perfusion studies (Figure 4).

**Figure 5:**
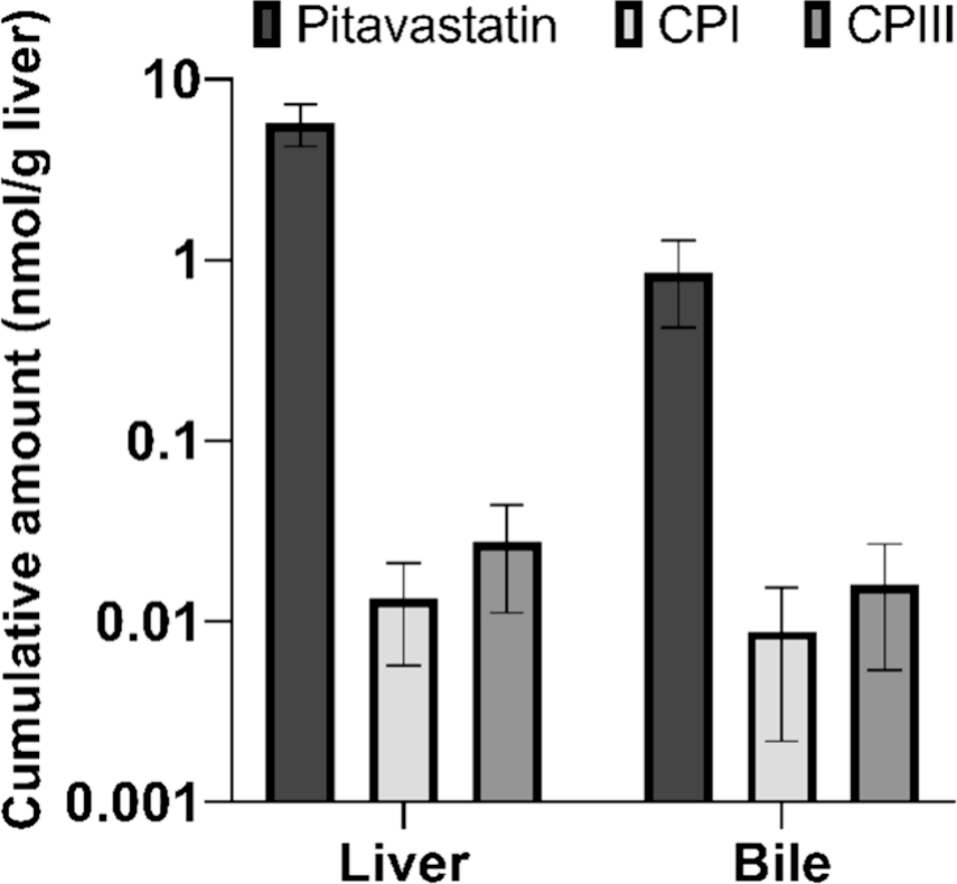
Amount of pitavastatin, and basal amounts of coproporphyrin I (CPI) and coproporphyrin III (CPIII) in the rat liver and bile after 60 min *in situ* isolated liver perfusion (*y*-axis is in log scale; *n* = 3, mean ± SD, liver weight= 7.83 ± 1.59 g).

After 60 min of recirculatory perfusion, 43% of the total pitavastatin dose was transported to the liver extracellular and intracellular space, whereas 6% of the dose was excreted in the bile. Both endogenous biomarkers were detected in samples (Figure 5). The results demonstrated a twofold higher abundance of CPIII than CPI in rat liver and bile (*p* > 0.05). The percentage of CPIII to the total amount of CPI and CPIII was 67.19 ± 4.28% and 64.96 ± 3.91% in the liver and bile, respectively.

## 4 Discussion

The *in situ* isolated perfused rat liver model (IPRL) is a multipurpose preclinical tool that can be designed to answer various pharmacokinetic questions including the mechanism of the hepatic drug transport and metabolism of exogenous and endogenous molecules. OATP transporters play a significant role in clearance mechanisms for many drugs and are involved in complex DDIs where substrates have an affinity to multiple drug-metabolizing enzymes and biliary efflux transporters.^41^ Endogenous biomarkers CPI and CPIII are transported by OATPs to the liver and undergo biliary excretion. CPs are suggested as informative markers to elucidate the mechanisms of complex drug–drug interactions involving various clearance pathways.^42^ Preclinical models are informative in elucidating the tissue distribution of drugs that are substrates of multiple hepatic drug transporters. The IPRL model, designed in a recirculatory/single-pass or retrospective manner, enables the evaluation of bidirectional drug transport and investigation of zonal differences in the liver. Moreover, this model can be used to explore the correlation between tissue concentrations of CPI/CPIII and Oatp substrate drugs in the presence or absence of perpetrator compounds, thus providing insights into hepatic drug transport mechanisms. The current experimental model can be applied for the new investigational drugs, which may cause interactions on Oatp transport with the simultaneous monitoring of biomarkers (CPI/III) using pitavastatin as the control substrate. In this study, an LC-MS method for the quantification of pitavastatin, CPI, and CPIII was developed. The validation of the method was limited to rat liver and bile matrices. Recirculatory perfusion did not allow quantification of CPs in the perfusate, due to dilution under the assay sensitivity. Although the investigation of CPs in liver tissue is important, the concentration of CPs in the perfusate, which mimics the systemic circulation, could support in assessing the function of the Oatp. However, we believe the developed method can be adapted in other samples and matrices (e.g., perfusate samples of single-pass IPRL, plasma samples from preclinical species, or human in clinical studies for monitoring of CPs in systemic circulation).

In the analytical model development, previously published methods for pitavastatin, CPI, and CPIII were evaluated integrally. A normal phase HPLC method by Kojima et al. has been developed for the investigation of pitavastatin in human plasma having a quantification limit of 0.001 μM (0.5 ng/mL) and a long run time (25 min). Other LC-MS/MS methods have been developed having faster analysis in 8 min^43^ and 2.1 min^44^ with higher sensitivity (0.0005 μM; 0.2 ng/mL). Pitavastatin is also a preferred model substrate in *in vitro* interaction studies. Menochet et al. used an LC-MS/MS method with a sensitivity of 0.005 μM to quantify pitavastatin in *in vitro* cell culture samples. However, the full validation of the method was not presented.^26^

Several HPLC^45,46^ and LC-MS/MS^16,47−50^ methods have been developed to quantify CPI and CPIII in plasma and urine samples. The sensitivity of these methods was similar, and the common choice was C18 and C18 pentafluorophenyl-type stationary columns. Commonly the gradient elution method was preferred, and depending on the column type, different ratios of acetonitrile and water (with ammonium formate or formic acid) were used.^16,49,50^

To the best of our knowledge, there is no analytical method for simultaneous analysis of pitavastatin and CPs. Although a comparative study^16^ using both OATP substrates (atorvastatin, fluvastatin, pitavastatin, and rosuvastatin) and biomarkers (bilirubin, glycochenodeoxycholate-3-sulfate, and CPI) has been performed to investigate OATP activity, drug substrates and endogenous biomarkers were analyzed separately with different methods with variations.

The extraction of CPI/III from biological materials is crucial. Takehara et al.^16^ used liquid extraction after adding 12 M formic acid to the plasma in a 2:1 ratio, while Njumbe et al.^49^ studied the protein precipitation with acetonitrile, solid extraction, and liquid extraction with acidified ethyl acetate and MTBE and optimized a solid phase extraction method.

Considering the multiple applications of endogenous biomarkers in drug development, the fit-for-purpose approach should be followed in the analytical method development.^50^ In this study, CPI and CPIII were analyzed in liver tissue homogenate and bile samples and the recovery of both isomers was obtained by using the triple extraction with acidified MTBE.

Due to the sixfold lower CPIII concentration than CPI in humans, Takehara et al.^16^ ignored the CPIII peak, which was obtained without clear separation from CPI. However, in rats, CPIII was found more dominant in serum, liver, bile and feces with an 88.4% ratio of CPIII to the total CPI and CPIII in bile.^22^ Thus, the analytical method provided the separation of CPI and CPIII, which also allowed simultaneous quantification of pitavastatin.

Our study confirmed the higher basal abundance of CPIII in both the liver and bile (Figure 5). As the perfusate samples were highly diluted, CPI and CPIII could not be detected in endogenous levels, which makes the evaluation of the Oatp-mediated transport of CPs challenging. The system design may be altered (e.g., single-pass perfusion) to be able to capture the basal levels of CPs in perfusate samples. Even so, the IPRL method provides valuable information for understanding the basal and time-dependent levels of CPs.

Expressed drug transporters and their activities differ between rats and humans. Additionally, interspecies differences in the renal and biliary excretion of CPs limit the translation of CP data from preclinic species to humans.^22^ However, due to the limitation of liver biopsy and bile collection in clinical studies, preclinical models can be utilized to evaluate the hepatic distribution of endogenous and exogenous biomarkers. By the IPRL preparation, pharmacokinetic DDIs between Oatp substrates in the absence/presence of inhibitors (e.g., rifampicin, cyclosporine) can be evaluated while monitoring the CPI/III levels in the liver and bile.

Since Oatp-mediated PK and DDI studies are important to evaluate the risk of clinical DDIs and require the use of a control model substrate (i.e., pitavastatin), the developed and validated LC-MS method in the present study can be adapted to biological matrices of interest (e.g., plasma) and utilized in future studies allowing simultaneous analysis of pitavastatin, CPI, and CPIII.

## 5 Conclusions

An LC-MS method for the simultaneous quantification of pitavastatin, CPI, and CPIII in rat liver and bile was developed and validated according to the ICH M10 guideline. All validation parameters showed that this method is applicable for further *in situ* isolated perfused rat liver preparations investigating the Oatp-mediated pharmacokinetics and DDIs.

## Supporting Information Available

(Supplement 1) The system suitability and (Supplement 2) linearity of the method (PDF)
