# Supplementary material for: An Isolated Perfused Rat Liver Model: Simultaneous LC-MS Quantification of Pitavastatin, Coproporphyrin I, and Coproporphyrin III Levels in the Rat Liver and Bile
Source: ACS Omega. 2024 Apr 18;9(17):19250–60. doi: 10.1021/acsomega.4c00109 (PMC11064166; doi:10.1021/acsomega.4c00109)
Supplement: Supplementary file 1 — ao4c00109_si_001.pdf [file ao4c00109_si_001.pdf]

Supporting Information

An isolated perfused rat liver model: Simultaneous LC-MS quantification of pitavastatin, coproporphyrin I and coproporphyrin III levels in the rat liver and bile

Nihan IZAT[1], Ozan KAPLAN[2], Mustafa CELEBIER[2], Selma SAHIN[1,*]

[1]Department of Pharmaceutical Technology, Hacettepe University, Faculty of Pharmacy, Ankara, Turkey

[2]Department of Analytical Chemistry, Hacettepe University, Faculty of Pharmacy, Ankara, Turkey

*Correspondence: sahin.selma@gmail.com

Supplement 1: The system suitability

Supplement 2: Linearity of the method

Supplement 1. The system suitability

Supplemental Table 1: The system suitability test results of the LC-MS method

	Matrix	Efficiency[a]	Symmetry factor[b]
**Pitavastatin**	Perfusate (0.075 µM)	34074	1.1
	Liver (0.250 µM)	6119	1.3
	Bile (2.500 µM)	14727	1.4
**CPI**	Liver (0.100 µM)	21134	1.4
	Bile (0.300 µM)	34661	1.3
**CPIII**	Liver (0.100 µM)	3522	1.1
	Bile (0.300 µM)	50121	1.5

[a]The number of theoretical plates (N) obtained in a gradient elution mode according to the formula N=16 (retention time/peak width)^2; b Symmetry factor (tailing factor, Tf) was calculated using the formula Tf = (a+b)/2a where a is the distance from the starting edge of the peak to the peak midpoint measured at 10% of peak height, b is the distance from the peak midpoint to the tailing edge of the peak measured at 10% of peak height.

Supplement 2 Linearity of the method

A

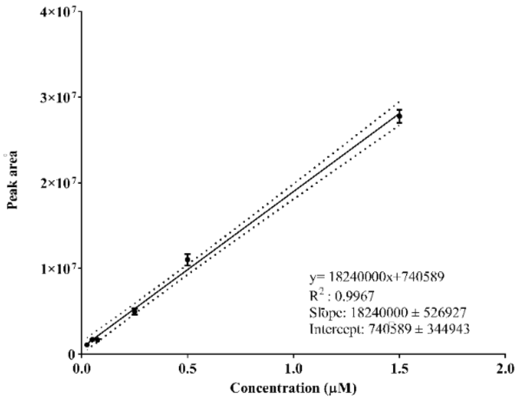

B

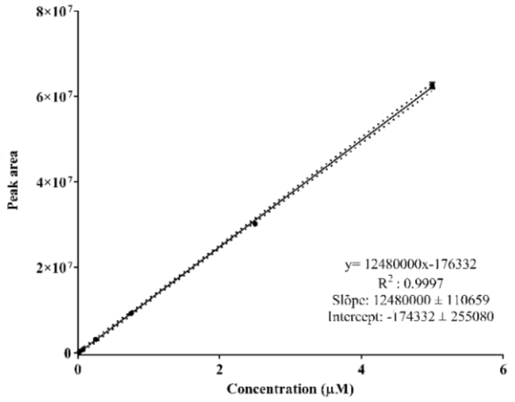

C

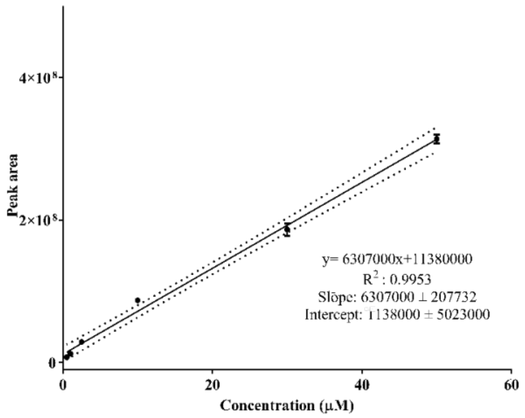

Supplemental Figure 1. The linearity of the method quantifies pitavastatin in the perfusate (A), liver (B) and bile (C) (mean ± SE; n = 6). X and Y-axis represent the concentration (μM) of pitavastatin and the peak area, respectively. The calibration curve and 95% confidence intervals are represented by solid lines and dotted lines, respectively. Calibration curve parameters (calibration equation, determination coefficient (R^2), slope ± SE, intercept ± SE) are given in the figure.

A

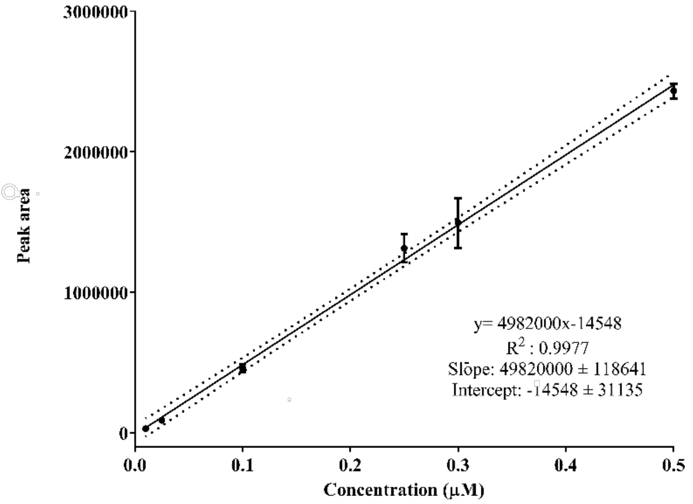

B

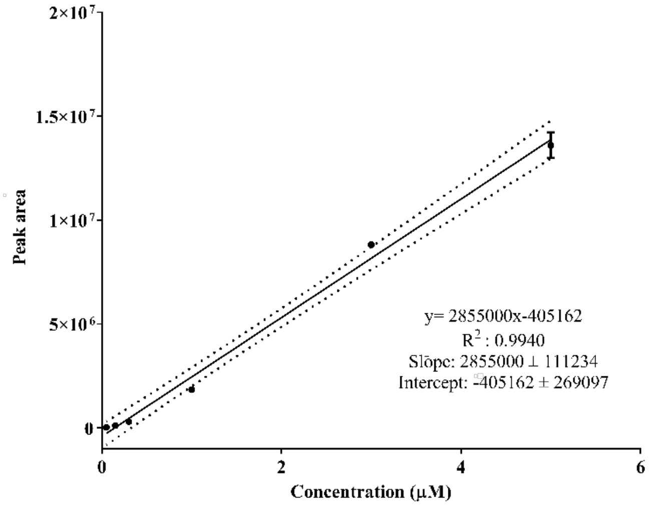

Supplemental Figure 2. The linearity of the method quantifies coproporphyrin I (CPI) in the liver (A) and bile (B) (mean ± SE; n = 6). X and Y-axis represent the concentration (µM) of CPI and the peak area, respectively. The calibration curve and 95% confidence intervals are represented by solid lines and dotted lines, respectively. Calibration curve parameters (calibration equation, determination coefficient (R^2), slope ± SE, intercept ± SE) are given in the figure.

A

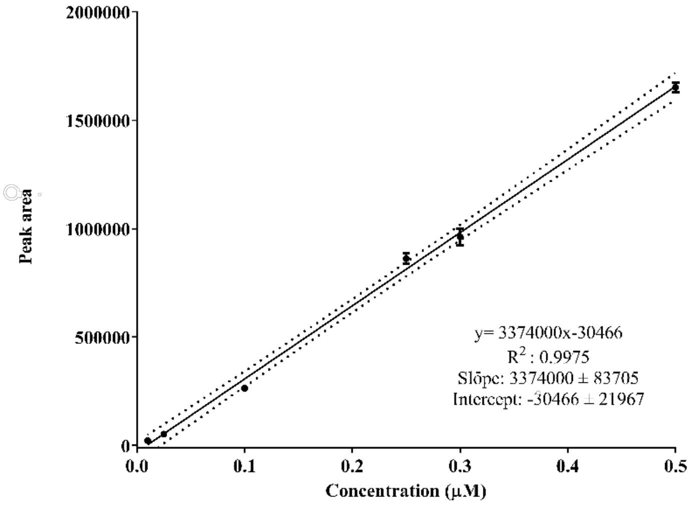

B

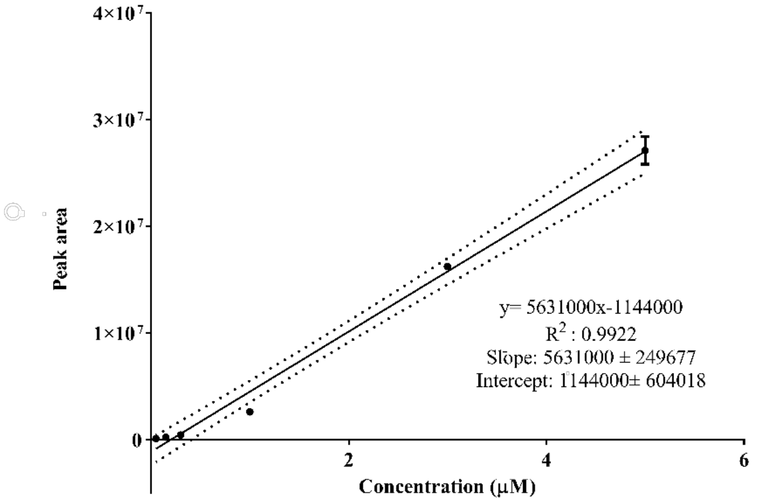

Supplemental Figure 3. The linearity of the method quantifying coproporphyrin III (CPIII) in the liver (A) and bile (B) (mean ± SE; n = 6). X and Y-axis represent the concentration (μM) of CPIII and the peak area, respectively. The calibration curve and 95% confidence intervals are represented by solid lines and dotted lines, respectively. Calibration curve parameters (calibration equation, determination coefficient (R^2), slope ± SE, intercept ± SE) are given in the figure.
